# ICT, health expenditures, and healthy life expectancy: empirical evidence from the Sub-Saharan African states

**DOI:** 10.3389/fpubh.2025.1664819

**Published:** 2026-01-12

**Authors:** Muhammet Ali Köroğlu, Yilmaz Bayar

**Affiliations:** 1 Department of Social Work, Faculty of Economics and Administrative Sciences, Usak University, Usak, Türkiye; 2 Department of Public Finance, Bandirma Onyedi Eylül University, Bandirma, Türkiye

**Keywords:** health expenditures, healthy life expectancy, ICT, out-of-pocket expenditures, panel econometrics, Sub-Saharan African countries

## Abstract

**Background/Objectives:**

Life expectancy (LE) is one of the most commonly used measures to describe the health of a population, which is crucial for achieving progress in economic growth and development. Therefore, identifying the drivers of LE is critical for designing effective health policies.

**Methods:**

This article investigates the interplay among indicators of health expenditures, the index of information and communication technologies (ICT), healthy life expectancy at birth (HALEB), and healthy life expectancy at age 60 (HALE60) in the Sub-Saharan African countries over the 2000–2021 period by means of robust causality and regression approaches.

**Results:**

The results of the causality analyses reveal a bidirectional causality among indicators of health expenditures, ICT, HALEB, and HALE60 at the panel level. However, the causal nexus among indicators of health expenditures, ICT, and healthy life expectancy differs among the Sub-Saharan African countries. Additionally, the consequences of the regression analysis unveil a positive effect of indicators of health expenditures and ICT development on HALEB and HALE60.

**Conclusion:**

The results suggest that both health expenditures and ICT development are significant factors influencing healthy life expectancy.

## 1 Introduction and background

Life expectancy (LE) is one of the important indicators of overall public health, and many institutional, economic, environmental, and social factors can affect life expectancy. In addition, increases in health expenditure and quantitative and qualitative improvements in health services are among the factors leading to the extension of life expectancy. However, the rate at which this extended life span is spent in good health is also important. From the individual perspective, the extended life expectancy can cause other family members to provide care for older adults in case of insufficient income of the retired persons (1). From the social perspective, extra economic costs can result from the sustainability of social security systems and the increases in labor dependency rates (2).

In this context, the effects of declines in mortality rates on the quality of life have become an important topic of discussion within the context of healthy life expectancy (HALE) (3). HALE is based on mortality and disability estimates of a population (4) and tries to reveal whether the current increases in life expectancy (LE) bring about disabilities, chronic diseases, or addictions (5). HALE has also increased together with LE all over the world. Life expectancy at birth (LEAB) was 66.8 years in 2000 and became 73.1 years in 2019. In addition, HALEB increased to 63.5 years from 58.1 between 2000 and 2021 (6). However, increases in both LEAB and HALEB decreased during the COVID-19 epidemic (6). In 2021, LEAB and HALEB were 71.4 and 61.9 years, respectively. Globally, LE at age 60 was 19.6 years for both sexes, while HALE60 was 14.7 years (6). However, increases in LEAB and HALEB remarkably differ in all countries, mainly due to the variations in the socio-economic levels of the countries. In this regard, Africa is one of the most disadvantaged regions in the world. LEAB in Africa was 63.6 years in 2021 (6), and life expectancy at age 60 was 16.7 years for both sexes in 2021 (6). HALEB was 55.2 years for both sexes in 2021, and it was 54.6 years for men and 55.7 years for women (6). HALE60 was 12.6 years for both sexes in 2021, and it was 12.2 years for men and 13 years for women (6).

In this regard, the health production function is an important source of information about the health status of a country and indicates the relationships between health inputs and outputs. Health-related inputs can include public and private health expenditures, environment, lifestyle, and genetic factors. Health outcomes can also be health measures, such as years of life lived with disability or healthy life expectancy (7). This research is based on Grossman's (8) health model, which suggests a model of the demand for good health. In the model, Grossman (8) bases his argument on the idea that individuals possess a stock of health from the beginning of their lives, which depreciates with aging but can be increased through health-related investments. In this respect, it considers health as a capital that is subject to genetic factors and age-related wear and tear (8). However, Grossman (9) revisits this model by focusing on the causality between education and good health and demonstrates that health production can vary depending on many different variables beyond medical care availability.

In conclusion, increases in healthcare expenditures and improvements in medical technologies are expected to positively impact the health production function within the framework of the health demand model. Health expenditures and out-of-pocket expenditures are also effective in reducing morbidity rates, infant mortality rates, and preventing and reducing epidemic diseases (10). Furthermore, information and communication technologies (ICT) have begun to be extensively used due to the increasing importance of advanced technological developments and communication. The widespread use of ICT also affects health conditions and indicators (11). Therefore, the efficient utilization of ICT in both preventive health services and the treatment of diseases is increasing. The use of health technologies for disadvantaged groups, such as the older adults and the disabled, and investments in this field are also increasing (12, 13). Therefore, ICT is also a significant driver of LE.

In this respect, many researchers have studied the factors behind improvements in LE in recent years (11, 14–19). However, only a limited number of studies have explored the factors underlying HALE in the literature. Therefore, this study would be one of the first empirical studies analyzing the nexus among various health expenditures, ICT, and HALE. Furthermore, the researchers have usually focused on the economic, social, environmental, and technological factors of LE. However, this study employs a two-way analysis to examine the effect of improvements in HALE on health expenditures and ICT development. The third empirical contribution of the study is to conduct the analyses using the sample of 44 Sub-Saharan African (SSA) countries, given the limited empirical studies on the determinants of HALE in the SSA countries. In this regard, the results of the study highlight that both indicators of health expenditures and ICT development are significant causes of HALEB and HALE60 in the SSA countries. However, this interaction seems insignificant in some SSA countries, mainly resulting from insufficient health and ICT infrastructure, prevalent income inequalities, and insufficient digital health literacy. In conclusion, these results would be useful for African health practitioners and policymakers to design and implement health policies.

The study consists of six sections. The introduction section presents the purpose and general theoretical background of the study. The second section includes the empirical literature review. In the third section, the dataset and methods are introduced. In the fourth section, the empirical analysis of the study is carried out, and the results are discussed in the fifth section. The study ends with a section including conclusions, limitations, and policy recommendations.

## 2 Literature review

The economic, social, and environmental determinants of longevity and health have been frequently discussed in the literature. Factors such as income level, out-of-pocket expenditures, and public health expenditures, level of access to food, and treatment opportunities are documented as the economic factors underlying LE (20–26). Demographic factors, such as sex and marital status, and lifestyle factors, such as tobacco and alcohol use, are among the social drivers of LE (16, 27–30). Environmental drivers of LE include factors such as a safe living conditions, productive environments, welfare level, and healthy urbanization (31–36).

The empirical studies have usually studied the effect of health-related indicators and ICT on LEAB. Therefore, this study investigates the bilateral interplay between health expenditures, out-of-pocket expenditures, ICT, HALEB, and HALE60 differently from the existing empirical literature.

### 2.1 Health expenditures and HALE

In the literature, the studies have usually focused on the national and regional estimates of HALE (37–42). On the other hand, some studies have examined the effect of sex, socioeconomic status, education level, ethnicity, and subgroups of the population on HALE (43, 44). In addition, some studies have been conducted to identify the determinants of HALE at the country and regional level. In this context, Robine and Ritchie (5) explored the factors underlying population health using the Sullivan method in the USA, Canada, England, and mainland Europe. The findings of the study revealed that significant differences in LE and disability-free LE between the richest and poorest income groups and health inequalities were particularly prevalent across the social groups. Furthermore, health expenditures and preventive health programs had a positive effect on reducing disability rates and increasing HALE. Therefore, the balanced distribution of public health expenditures is effective in improving public health, eliminating health inequalities, and increasing HALE, especially in developed countries.

Mathers et al. (45) examined HALE in 1999 in 191 WHO member countries within the scope of the Global Burden of Diseases, Injuries and Risk Factors (GBD) study. They based their study on the analysis of the results of 60 health surveys conducted around the world and estimates of 109 causes of disease and injury, and calculated HALE for each sex using the Sullivan method. The country with the highest average HALEB was Japan, with 74.5 years, while the 10 SSA countries where the HIV-AIDS epidemic was widespread had the lowest HALEB, with 35 years. The study also concluded that HALE increased faster than total LE in parallel with the increases in health expenditures per capita. This result revealed that the decrease in mortality rates was due to the decrease in disability rates. The study also found that total LE is affected by different variables such as the economic development level of countries, inequalities in access to health services, epidemics, regional wars, migration, or natural disasters, and varies at the country level.

In their study, Salomon et al. (46) calculated HALE for 187 countries for the years 1990–2010 using the 2010 inputs of the GBD research. As a result of the study, they found that HALE varied among the countries, and the number of years spent with disability increases due to the increases in LE in most countries. In this context, HALE increased in 19 of the 21 regions examined. However, due to the HIV/AIDS epidemic in southern Sub-Saharan Africa and the major earthquake in the Caribbean in 2010, LE decreased significantly. The findings of the study also revealed that years spent with disability impose a significant burden on health expenditures and health planning.

Corris et al. (47) uncovered that HALE was lower and health inequalities were worsening, especially in the North East compared with the other parts of England. They also found that health inequalities, the provision, access, and quality of health services, as well as reduced health expenditures due to austerity policies, income inequality, and increasing child poverty are the main factors affecting HALE. The findings of the study also revealed that the negative impact of the unequal distribution of health services and health expenditures on regional health outcomes in a European country with a high level of economic development.

Cao et al. (48) examined the relationships between socioeconomic, environmental, disease-related factors, and LE, HALE, and GAP (difference between LE and HALE) based on data from 195 countries for the years 1995–2017 using multiple linear regression analysis. As a result of the study, they found that LE and HALE increased in 186 of 195 countries, and the increase in LE and HALE was related to economic growth, welfare policies, health expenditures, coverage of health services, use of health technologies, effectiveness of practices aimed at controlling and preventing disability, and mortality rates. They suggested that improving the public health infrastructure, increasing the number of qualified health personnel, and providing free access to health services and quality health services are required, especially for underdeveloped countries. Furthermore, establishing preventive health services systems and taking measures for a healthy lifestyle can reduce the risks of chronic diseases and increase HALE for the developed countries.

Based on the related theoretical and empirical literature, the first two hypotheses of this study are specified as follows:

HP1: There exists a mutual interplay among per capita health expenditures and out-of-pocket expenditures and HALEB/HALE60.
HP2: Per capita health and out-of-pocket expenditures positively impact HALEB and HALE60.

### 2.2 ICT and HALE

ICT can expand the use of healthcare services across the general population, improve the quality of services provided, and reduce costs by making health systems more efficient (49, 50). ICT not only affects HALE but also affects healthy aging. In old age, ICT can be used to reduce disability and social isolation and to achieve the goal of a healthy, active, and productive life (51). In this regard, ICT enables the use and sharing of information through independent of time and place. Therefore, it can contribute to the collection, storage, and functional reuse of patient information (52, 53). ICT can also accelerate the healing process by ensuring patients' participation in decision-making processes (54). In addition, it can help achieve many important goals, from disease prevention to awareness raising, through ICT-supported educational programs and applications (55).

The Theory of Reasoned Action, Technology Acceptance Theory, Displacement Theory, and Uses and Gratifications suggest a relationship between digital technology use and health at theoretical considerations (14, 56). However, in the associated empirical literature, no studies about the nexus between ICT and HALE have been found. In this respect, this study aims to contribute to the empirical literature by investigating this nexus between two variables. However, the studies have usually investigated the nexus between ICT and LEAB and revealed that different indicators related to ICT mostly have had a positive effect on LEAB (57).

In the literature, Wu and Raghupadhi (58) used panel data analysis for five countries with different income levels based on World Development Indicators data for the period 2000–2008, and the findings of the study revealed that the use of ICT in education and preventive healthcare strategies was effective in increasing LE and reducing adult mortality rates. In another study, Raghupathi and Raghupathi (59) examined the relationship between ICT and public health at the country level within the framework of the health analytics approach and determined a positive relationship between ICT factors and LE.

Alzaid et al. (60) analyzed the effects of sharing medical information on the Internet on public health through regression analysis. Their outcomes revealed that the information production and sharing functions of the Internet contributed to the increases in LE. Additionally, the Internet also acted as a mediator between LE and economic growth. Mimbi and Bankole (61) analyzed the nexus between ICT usage and LEAB in 27 African countries for the period 1998–2007 using a multi-method approach and found a positive effect of ICT usage on LEAB and a negative effect of ICT on mortality in infancy. They demonstrated the effectiveness of ICT use in improving national healthcare systems and emphasized the need to invest in health systems in all African countries, especially in light of the Ebola outbreak in West Africa.

Majeed and Khan (62) studied the impact of ICT indicators on LEAB and infant mortality in 184 countries over the period of 1990–2014 and revealed that ICT use and digitalization positively affected LEAB. In line with the findings of the study, they suggested increasing digital inclusion in countries' future health policies. Afroz et al. (63) examined the impact of ICT on population health proxied by LEAB and infant mortality rates in Malaysia between 1993 and 2017 using the ARDL approach. Their findings showed that increasing health literacy, facilitating access to health-related information and health services, and supporting health systems with digital technologies had an improving effect on public health. Byaro et al. (64) also investigated the impact of Internet access on health outcomes in 48 SSA countries based on health promotion theory. The findings of the study, in which the generalized quantile regression approach was used, determined that for the period 2000–2020, Internet use increased longevity and reduced both infant and under-five mortality rates. The findings of the study revealed that the widespread use of smartphones and computers and the development of Internet infrastructure had a positive impact on LE in Sub-Saharan African Countries. In another recent study, Bétila (65) examined the relationship between public health expenditures, ICT development, and health outcomes in 48 African countries between 2000 and 2018 using regression. The results of the study showed that ICT had a negative impact on mortality rates and a positive impact on LEAB when associated with public health expenditures. Megbowon and David (66) examined the impact of ICT development on health gaps in 38 countries between 2010 and 2018. The findings of the study revealed that ICT is a stimulating factor in exceeding the international health target of 60 years LEAB in Africa. In addition, the results of the study showed that the Internet component of ICT had a positive effect on improving both individual and community health. Kouton et al. (56) also examined how ICT development impacted health outcomes for 35 countries in Africa between 2000 and 2016. The findings of the study revealed that ICT development in a free economy positively affected health outcomes. Adeola and Evans (67) examined the causality between ICT and health in Africa during the period 1995–2015, and the findings of the study revealed that ICT use promoted health, which in turn encourages ICT use.

Unlike these studies, Bayar et al. (68) investigated the bidirectional causality between ICT and LE in emerging market economies over the years 1997–2020 by means of an asymmetric causality test and unveiled that mobile subscriptions and Internet usage had a significant effect on LE. Furthermore, Morawczynski and Ngwenyama (69), Hajli and Featherman (70), Dutta et al. (71), and Rahman and Alam (72) uncovered a positive influence of ICT on LE. Contrary to these studies, some studies found a negative or insignificant nexus between ICT indicators and LE. As a matter of fact, inappropriate use of ICT can negatively affect both physical and psychological health. Visual disturbances and dry eyes, back, neck, and wrist pain (73), physical problems due to a change in leisure activities and inactivity (74), and overweight and obesity (75) are among the most negative problems resulting from ICT usage. Inappropriate use of information and communication technologies can also lead to psychological addictions (76).

In this context, Shao et al. (55) analyzed the nexus between ICT and public health service delivery in a panel of 141 countries for the period 2012–2016 with the regression method and the Sobel test and identified the facilitating effect of ICT on public health service delivery. They found that ICT was effective in reducing under-five and maternal mortality rates and adolescent pregnancies. However, they also found that ICT had no significant effect on LE. Wang et al. (14) studied the interaction between Internet and mobile phone usage and LEAB in the low-income countries for the period 2000–2017 and found that Internet and mobile phone use positively affected health and led to an increase in LEAB, while increased phone-based ICT use negatively affected health and led to a decrease in LEAB. Finally, Ilikkan Özgür et al. (77) investigated the impact of ICT indicators on health outcomes in BRICS countries and Turkey for the years 1990–2018 and found that all ICT indicators had a negative impact on LE. They revealed that increasing the comprehensiveness of ICT, expanding its correct use to improve communication between patients and health systems, and increasing health literacy could have a positive impact on public health.

Based on the related theoretical and empirical literature, the last two hypotheses of this study are formed as follows:

HP3: There exists a mutual interplay between ICT and HALEB/HALE60.
HP4: ICT development positively impacts HALEB/HALE60.

## 3 Data and methods

This study explores the effect of health expenditures per capita, out-of-pocket expenditures per capita, and ICT index on HALEB and HALE60 by means of causality and regression approaches. The variables utilized in the applied analyses are exhibited in Table 1. In this regard, healthy life expectancy is represented by HALEB and HALE60, and both variables are obtained from the WHO (6). On the other hand, health expenditures are proxied by current health expenditure per capita and out-of-pocket expenditure per capita and provided by the World Bank (78, 79). Both current health expenditures per capita and health expenditure through out-of-pocket payments per capita are in terms of international dollars based on purchasing power parity. Finally, development level of ICT is represented by ICT index of UNCTADStat (80), which is calculated by using the series of fixed broadband subscriptions (per 100 people), fixed telephone subscriptions (per 100 people), individuals using the Internet (% of population and mobile cellular subscriptions (per 100 people) from ITU World Telecommunication, and Secure Internet servers (per 1 million people) from Netcraft (netcraft.com), and World Bank population estimates through principal components analysis. The loadings for ICT for each factor are respectively 0.93, 0.82, 0.77, 0.92, and 0.91, and the variance explained is 77% [see UN (81) for detailed information about this index].

**Table 1:** Variables utilized in the econometric analyses.

**Variables**	**Data definition**
HALEB	Healthy life expectancy at birth (years)
HALE60	Healthy life expectancy at 60 (years)
HEALTH	Current health expenditure per capita (current international $; purchasing power parity)
POCKET	Out-of-pocket expenditure per capita (current international $; purchasing power parity)
ICT	ICT index

The sample of the research consists of 44 SSA economies (Angola, Benin, Botswana, Burkina Faso, Burundi, Cabo Verde, Cameroon, Central African Republic, Chad, Comoros, Congo, Dem. Rep., Congo, Rep., Cote d'Ivoire, Equatorial Guinea, Eritrea, Eswatini, Ethiopia, Gabon, Gambia, Ghana, Guinea, Guinea-Bissau, Kenya, Lesotho, Liberia, Madagascar, Malawi, Mali, Mauritania, Mauritius, Mozambique, Namibia, Niger, Nigeria, Rwanda, Sao Tome and Principe, Senegal, Seychelles, Sierra Leone, South Africa, Tanzania, Togo, Uganda, and Zambia except Somalia, South Sudan, Sudan, and Zimbabwe) due to data non-availability considering the classification of World Bank (82), and the study period is designated as 2000–2021 because health indicators and HALE indicators are available between 2000 and 2021.

The econometric analyses are carried out using the Stata 17.0 and EViews 12.0 According to the summary statistics presented in Table 2, the averaged values of HALEB, HALE60, HEALTH, POCKET, and ICT are 52.063 years, 12.132 years, USD 215.987, USD 65.052, and 17.226, respectively. However, health expenditures per capita and out-of-pocket expenditures per capita significantly change among the SSA countries, while the ICT index and healthy life expectancy at birth (HALEB) and at 60 years show a moderate variation among these countries over the 2000–2021 period.

**Table 2:** Summary statistics of the series.

**Variables**	**Averaged values**	**Std. Dev**.	**Min**	**Max**
HALEB	52.063	5.895	37.04	67.09
HALE60	12.132	1.421	8.27	17.14
HEALTH	215.987	268.439	6.343	1,729.844
POCKET	65.052	80.889	3.064	663.035
ICT	17.226	12.816	1	74.534

The causality relationship between health expenditures, out-of-pocket expenditures, ICT, HALEB, and HALE60 in the SSA countries is analyzed by the causality tests of Emirmahmutoglu and Kose (83) and the Juodis et al. (84). The causality tests only investigate whether one time series is useful for forecasting another time series and indicate the direction of the relationship between the two series (85). The E-K causality test is a developed version of Toda and Yamamoto's (86) causality for panel datasets with CD and heterogeneity. The predicted VAR (vector autoregression) model for each country is as follows:


$$\begin{array}{l} y_{it}=\delta_{i}+A_{1i}y_{i\left(t-1\right)+\ldots +}A_{pi}y_{i\left(t-pi\right)+\ldots +}A_{\left(p+d\right)i}y_{i\left(t-pi-di\right)}+u_{i,t} \end{array}$$ (1)

where *y*_*it*_ is an endogenous variable, δ_*i*_ is a fixed-effects vector comprising p dimensions, p_i_ is the optimal lag length, and d_i_ is the highest integration level of the series included in the analyses. Furthermore, this causality test makes it possible for the lag length to become different for each country and thus decreases the loss of long-term information resulting from modeling the series with level values (83). However, as the cross-section of the dataset increases, the possibility of attaining large values for some cross-sections also increases, and, in turn, over-rejection of the null hypothesis for small T values can be possible (87). In this case, the causality test of Juodis et al. (84) rests upon the Split Panel Jackknife procedure can lead the Wald statistic to have good size and power properties for datasets with large N and moderate T dimensions.

Finally, the regression method is also applied to disclose the influence of indicators of health expenditures and the ICT index on HALEB and HALE60. In this regard, the presence of serial correlation and heteroscedasticity violating regression assumptions caused us to make a selection among the Parks–Kmenta estimator, the Driscoll–Kraay estimator, and the Beck–Katz estimator. However, the Driscoll–Kraay estimator rather than the Parks–Kmenta estimator is preferred because cross-sectional dimension of the dataset is greater than time dimension of the dataset (39).

## 4 Results

In the empirical section of the study, the entity of collinearity in the model is first examined by means of correlation and VIF (variance inflation factor) methods. In this context, the correlation matrix among the explanatory variables and VIF scores is calculated and introduced in Table 3. A VIF value greater than 5 or 10 points out a collinearity problem (88). In conclusion, the low VIF and correlation values refer to the non-availability of the collinearity problem in the models.

**Table 3:** Correlation matrix.

**Variables**	**HEALTH**	**POCKET**	**ICT**	**VIF**
HEALTH	1	0.398^**^	0.345^**^	3.102
POCKET		1	0.198^**^	2.574
ICT			1	2.098

^**^Indicates statistical significance at the 5%.

Furthermore, the presence of endogeneity in both models is analyzed using the Durbin–Wu–Hausman test, and the results are presented in Table 4. The null hypothesis (presence of exogeneity) is accepted in light of the *p*-values. Thus, the endogeneity problem does not exist in both models.

**Table 4:** The results of the Durbin–Wu–Hausman test.

**Model-1**		**Model-2**	
**The residual of independent variables**	***p*** **values**	**The residual of independent variables**	***p*** **values**
HALEB_t − 1_	0.143	HALE60_t − 1_	0.125
HEALTH	0.324	HEALTH	0.414
POCKET	0.256	POCKET	0.358
ICT	0.562	ICT	0.486

The presence of heterogeneity and CD is respectively examined by the delta tilde and Lagrange multiplier (LM) tests. In this regard, the test of LM_adj._, LM CD, and LM are conducted for both models, and their outcomes are exhibited in Table 5. The null hypothesis of CD is rejected because the probability values of these are lower than 1%, and the presence of CD is uncovered. Then, a homogeneity test of delta tilde is conducted, and its outcomes are presented in Table 5. The null hypothesis of homogeneity is rejected at 1% level, and the presence of heterogeneity is disclosed.

**Table 5:** Findings of CD and homogeneity tests.

**CD tests**		**Slope homogeneity tests**	
**Test**	**Test statistic**	**Test**	**Test statistic**
**Model-1**			
LM_adj._	74.49^***^	Delta	28.372^***^
LM CD	25.76^***^	Bias-Adj. Delta	32.276^***^
LM	2,462^***^		
**Model-2**			
LM_adj._	151.4^***^	Delta	24.717^***^
LM CD	43.61^***^	Bias-Adj. Delta	28.118^***^
LM	3,914^***^		

^***^Significant at 1%.

The presence of a unit root in the series of HALEB, HALE60, HEALTH, POCKET, and ICT should be specified for causality analysis. Therefore, the panel CIPS unit root test of Pesaran (89) is employed owing to the presence of CD, and its outcome is exhibited in Table 6. The outcomes unveil that the integration levels of HALEB, HALE60, HEALTH, POCKET, and ICT are one, demonstrating that all series are non-stationary at level, but seem stationary with their first difference values.

**Table 6:** CIPS test outcomes.

**Variables**	**Level**		**First differenced values**	
	**Constant**	**Constant**+ **Trend**	**Constant**	**Constant**+ **Trend**
HALEB	−0.031	0.146	−3.051^***^	−3.882^***^
HALE60	−1.867	−1.802	−2.915^***^	−4.051^***^
HEALTH	−0.319	−1.111	−4.668^***^	−4.789
POCKET	−1.092	−1.346	−3.959^***^	−4.173^***^
ICT	−0.408	−0.974	−3.533^***^	−3.983^***^

^***^Significant at 1%.

**Figure 1:**
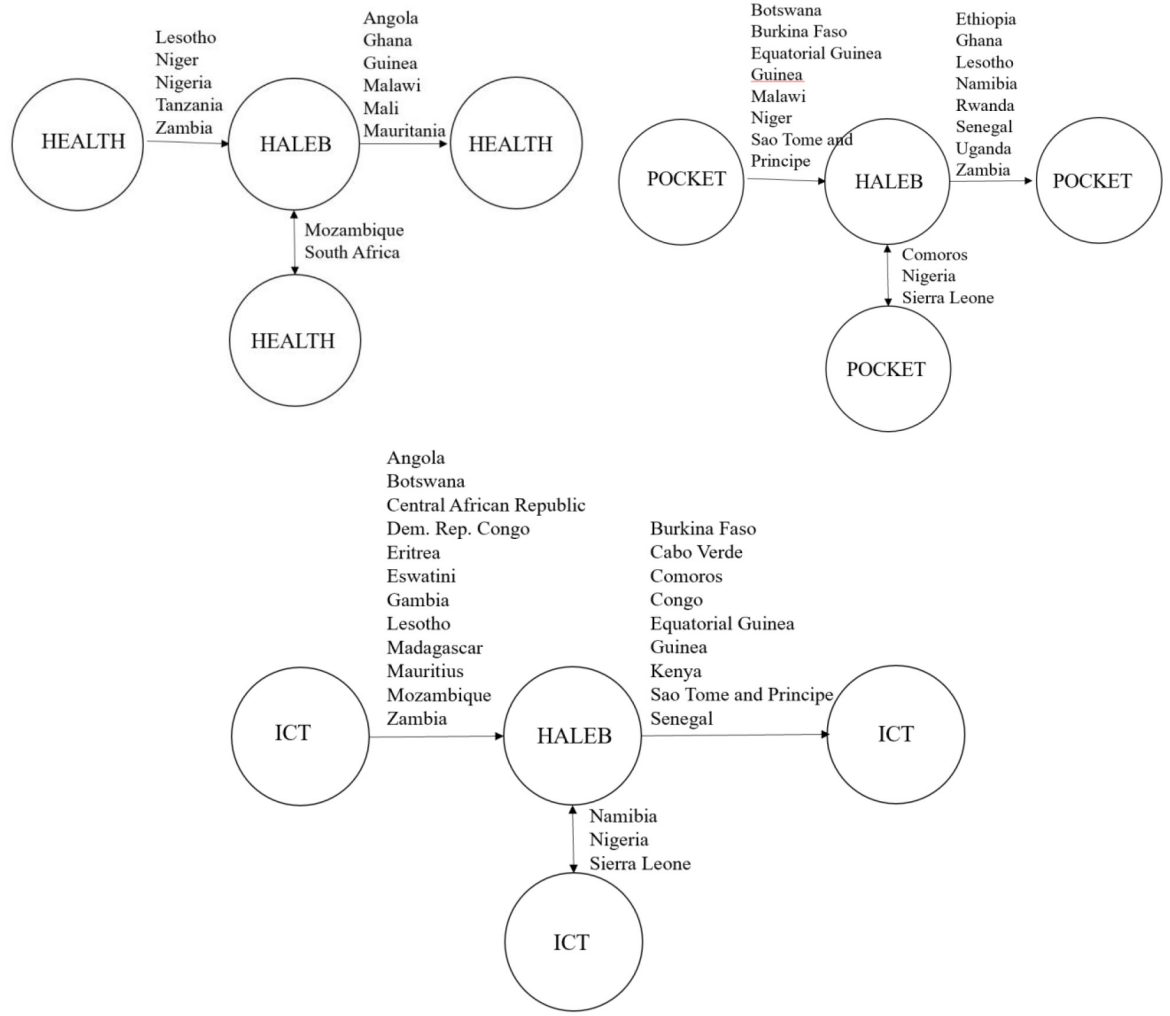
The results of the causality test among HEALTH, POCKET, ICT and HALEB. → indicates one-way causality from … to …. while ↔ indicates a two-way causality between two variables.

The causal interplay among HEALTH, POCKET, ICT, and HALEB is analyzed by means of the causality test of E-K (83), and then the Benjamin–Hochberg false discovery rate (FDR) procedure is performed through the obtained *p* values, and the final outcomes are introduced in Table 7. Normally, the null hypothesis is rejected for 11 of 44 countries for two-way causality between health expenditures and HALEB, but 8/44 remain significant after the FDR procedure for both causality analyses. The final results are reported in Table 7 and Figure 1. The panel-level results point out a bidirectional causality among health expenditures, out-of-pocket expenditures, ICT, and HALEB. However, the results of country-level analysis uncover that the causal nexus among these variables differs among the SSA countries. In this context, unidirectional causality from health expenditures to HALEB is observed Eritrea, Lesotho, Niger, Nigeria, Tanzania, and Zambia; unidirectional causality from HALEB to health expenditures occurs in Angola, Ghana, Guinea, Malawi, Mali, and Mauritania; and bidirectional causality between the two variables is found in Mozambique and South Africa.

**Table 7:** Results of causality analysis among HEALTH, POCKET, ICT, and HALEB.

**Countries**	**HEALTH↛ HALEB**	**HALEB↛ HEALTH**	**POCKET↛ HALEB**	**HALEB↛ POCKET**	**ICT↛ HALEB**	**HALEB↛ ICT**
Angola	0.321	9.655^**^	0.624	0.596	18.710^***^	0.214
Benin	0.294	1.344	0.017	0.028	0.014	0.007
Botswana	3.971	1.444	85.615^***^	1.197	31.827^***^	0.431
Burkina Faso	4.692	0.795	7.492^***^	0.314	3.122	12.990^***^
Burundi	0.218	0.871	0.077	1.629	0.481	0.278
Cabo Verde	3.057	0.186	2.570	6.375	0.247	55.457^***^
Cameroon	1.722	3.147	1.049	0.444	3.710	2.203
Central African Republic	0.178	0.464	0.987	0.382	6.220^**^	0.131
Chad	0.001	0.153	0.523	0.545	3.350	1.053
Comoros	1.492	0.039	10.666^**^	18.165^***^	3.024	7.860^**^
Congo	0.680	0.950	0.182	0.380	0.561	11.776^***^
Cote d'Ivoire	2.847	0.131	3.854	1.763	0.156	0.014
Dem. Rep. Congo	0.479	0.045	0.144	1.404	8.812^**^	0.954
Equatorial Guinea	2.244	2.538	6.309^**^	1.364	5.067	7.459^*^
Eritrea	10.234^**^	2.926	2.371	1.651	10.612^***^	2.430
Eswatini	3.596	4.391	7.227	1.839	6.352^**^	2.462
Ethiopia	2.700	0.452	3.802	8.650^**^	4.417	4.268
Gabon	2.135	3.030	1.888	3.260	4.740	1.350
Gambia	1.033	5.409	1.422	1.209	13.749^***^	1.526
Ghana	1.000	13.061^***^	3.141	19.652^***^	4.270	3.754
Guinea	1.871	11.108^**^	4.710^*^	1.342	1.031	5.148
Guinea-Bissau	0.009	0.140	4.392	0.764	1.856	3.619
Kenya	2.158	0.930	3.121	7.010	4.617	8.957^**^
Lesotho	11.111^**^	0.409	2.399	13.350^***^	35.066^***^	8.280
Liberia	0.617	0.067	3.045	0.984	1.571	0.457
Madagascar	4.515	1.205	2.031	0.011	18.039^***^	4.898
Malawi	0.119	15.219^***^	4.219^*^	1.070	0.110	2.189
Mali	0.242	33.684^***^	3.618	0.576	2.330	4.230
Mauritania	2.177	12.573^***^	0.382	0.043	1.331	3.427
Mauritius	2.274	4.771	0.663	4.961	14.792^***^	0.879
Mozambique	29.165^***^	15.327^***^	0.815	0.779	12.395^***^	3.682
Namibia	1.949	0.413	2.537	12.566^***^	119.360^***^	6.760^*^
Niger	4.734^*^	0.473	20.173^***^	0.524	0.151	1.456
Nigeria	12.573^**^	8.521	19.705^***^	11.390^***^	16.207^***^	10.793^**^
Rwanda	3.119	4.252	2.587	24.179^***^	1.057	3.137
Sao Tome and Principe	0.061	4.420	4.021^*^	2.831	2.247	14.110^***^
Senegal	1.362	0.667	3.423	13.294^***^	5.440	13.772^***^
Seychelles	1.266	1.256	1.719	1.513	0.829	3.051
Sierra Leone	5.832	0.839	10.314^**^	12.490^***^	8.695^**^	16.891^***^
South Africa	32.378^***^	8.736^**^	3.594	2.025	0.315	3.269
Tanzania	15.570^***^	1.376	1.464	0.240	3.032	0.002
Togo	0.616	1.530	0.316	1.403	0.942	3.252
Uganda	1.724	3.539	1.618	11.524^***^	0.350	2.216
Zambia	8.814^**^	1.906	2.809	11.500^***^	12.943^***^	2.309
Panel	179.782^***^	174.895	230.984^***^	187.142^***^	374.718^***^	225.615^***^

Optimal lags are determined by Akaike information criterion.

↛: X is not Granger cause of Y.

^***^, ^**^, and ^*^ significant at 1%, 5%, and 10%, respectively.

In a similar vein, a null hypothesis is rejected for 12 of 44 countries for causality from out-of-pocket expenditures to HALEB, but 10/44 stay significant after implementation of the FDR procedure. On the other hand, the null hypothesis is rejected for 15 of 44 countries for causality from HALEB to out-of-pocket expenditures, but 11/44 stay significant after implementation of the FDR procedure. The final results are reported in Table 6. In this regard, there exists a unidirectional causality from out-of-pocket expenditures to HALEB in Botswana, Burkina Faso, Equatorial Guinea, Guinea, Malawi, Niger, and Sao Tome and Principe, and a unidirectional causality from HALEB to out-of-pocket expenditures in Ethiopia, Ghana, Lesotho, Namibia, Rwanda, Senegal, Uganda, and Zambia and a bidirectional causality nexus between two variables in Comoros, Nigeria, and Sierra Leone.

Finally, the null hypothesis is rejected for 18 of 44 countries for causality from ICT to HALEB, but 16/44 stay significant after implementation of the FDR procedure. On the other hand, the null hypothesis is rejected for 13 of 44 countries for causality from HALEB to ICT, but 11/44 stay significant after implementation of the FDR procedure. In this regard, there exists a unidirectional causality from ICT to HALEB in Angola, Botswana, Central African Republic, Dem. Rep. Congo, Eritrea, Eswatini, Gambia, Lesotho, Madagascar, Mauritius, Mozambique, and Zambia, and a unidirectional causality from HALEB to ICT in Burkina Faso, Cabo Verde, Comoros, Congo, Equatorial Guinea, Guinea, Kenya, Sao Tome and Principe, Senegal, and a bidirectional causality between two variables in Namibia, Nigeria, and Sierra Leone.

The causal interplay among HEALTH, POCKET, ICT, and HALE60 is analyzed by means of the causality test of E-K (79), and then the FDR procedure is performed through the obtained *p*-values, and the final outcomes are introduced in Table 7. Normally, the null hypothesis is rejected for 17 of 44 countries for causality from health expenditures to HALE60, but 13/44 stay significant after the FDR procedure. On the other hand, a null hypothesis is rejected for 11 of 44 countries for causality from HALE60 to health expenditures, but 8/44 stay significant after the FDR procedure. The final results are reported in Table 8. The panel-level results indicate a bidirectional causality among health expenditures, out-of-pocket expenditures, ICT, and HALE60. In this context, there exists a unidirectional causality from health expenditures to HALE60 in Central African Republic, Comoros, Congo, Cote d'Ivoire, Eswatini, Namibia, Rwanda, South Africa, Zambia and unidirectional causality from HALE60 to health expenditures in Malawi, Mali, Mauritania, and Sao Tome and Principe, and bidirectional causality between two variables in Kenya, Mozambique, Nigeria, and Tanzania.

**Table 8:** Results of causal analysis among HEALTH, POCKET, ICT, and HALE60.

**Countries**	**HEALTH↛ HALE60**	**HALE60↛ HEALTH**	**POCKET↛ HALE60**	**HALE60↛ POCKET**	**ICT↛ HALE60**	**HALE60↛ ICT**
Angola	2.711	2.742	3.660	2.371	0.256	2.191
Benin	3.712	4.193	0.023	0.548	0.164	0.003
Botswana	1.791	0.143	36.260^***^	7.461^*^	12.332^***^	1.963
Burkina Faso	1.416	4.092	0.182	0.075	1.615	6.981^*^
Burundi	0.335	0.353	1.102	0.922	0.639	1.982
Cabo Verde	0.669	0.006	1.932	3.867	3.006	34.330^***^
Cameroon	0.723	3.991	0.415	3.716	17.961^***^	1.973
Central African Republic	41.221^***^	0.943	4.830	2.524	1.413	0.011
Chad	0.101	0.162	0.000	0.001	3.225	19.345^***^
Comoros	19.518^***^	5.314	4.477	12.089^***^	2.190	4.504
Congo	10.990^***^	4.095	0.721	3.281	0.380	1.148
Cote d'Ivoire	9.491^***^	1.399	3.750	0.138	6.901^*^	2.331
Dem. Rep. Congo	0.189	0.280	5.171	0.714	0.108	0.073
Equatorial Guinea	0.696	1.810	13.030^***^	0.212	4.450	0.684
Eritrea	2.165	1.171	0.473	0.231	50.419^***^	1.945
Eswatini	23.475^***^	1.146	8.724^**^	3.610	1.854	4.707
Ethiopia	1.064	0.280	2.755	6.699^*^	0.172	0.406
Gabon	0.367	2.240	0.386	0.913	0.013	1.780
Gambia	0.706	2.025	1.727	0.525	7.075^*^	16.538^***^
Ghana	5.214	2.316	0.374	0.001	0.235	0.890
Guinea	0.004	0.292	0.949	2.917	52.532^***^	5.980
Guinea-Bissau	0.718	1.354	5.137^*^	1.458	1.985	0.705
Kenya	8.044^**^	14.435^***^	15.080^***^	7.504^*^	18.087^***^	0.243
Lesotho	5.376	0.069	1.929	3.520	24.313^***^	25.930^***^
Liberia	3.448	0.111	4.429	3.292	0.714	1.138
Madagascar	4.231	2.920	1.575	0.051	11.355^***^	3.827
Malawi	0.573	7.890^**^	1.641	1.203	3.323	0.013
Mali	0.492	24.207^***^	1.725	0.090	2.509	1.466
Mauritania	2.903	13.703^***^	0.445	0.000	3.413	4.039
Mauritius	1.614	1.449	0.425	1.886	65.803^***^	9.372^**^
Mozambique	8.710^**^	27.990^***^	7.034^*^	16.787^***^	30.115^***^	105.186^***^
Namibia	10.414^**^	2.460	2.395	23.029^***^	9.289^**^	3.722
Niger	2.800	0.090	1.370	0.804	0.013	1.310
Nigeria	7.169^*^	9.474^**^	15.159^***^	14.189^***^	0.771	3.264
Rwanda	14.174^***^	1.424	1.353	5.514^*^	1.509	4.210
Sao Tome and Principe	3.194	23.355^***^	2.537	0.974	4.859	6.481^*^
Senegal	0.334	2.908	0.347	0.428	3.078	9.045^**^
Seychelles	1.285	1.783	0.874	1.937	1.236	2.273
Sierra Leone	3.922	4.784	10.668^**^	17.332^***^	4.476	4.624
South Africa	18.452^***^	2.102	2.901	6.343^*^	12.145^***^	3.302
Tanzania	10.055^***^	5.302^*^	2.109	0.950	12.902^***^	2.185
Togo	4.566	3.265	1.259	1.561	0.737	0.001
Uganda	3.146	3.273	4.002	6.282^*^	6.588^*^	3.063
Zambia	35.995^***^	1.522	3.023	19.313^***^	1.435	2.209
Panel	268.079^***^	187.536^***^	172.590^***^	178.608^***^	357.349^***^	279.585^***^

Optimal lags are determined by Akaike information criterion.

↛: X is not Granger cause of Y.

^***^, ^**^, and ^*^ significant at 1%, 5%, and 10%, respectively.

**Figure 2:**
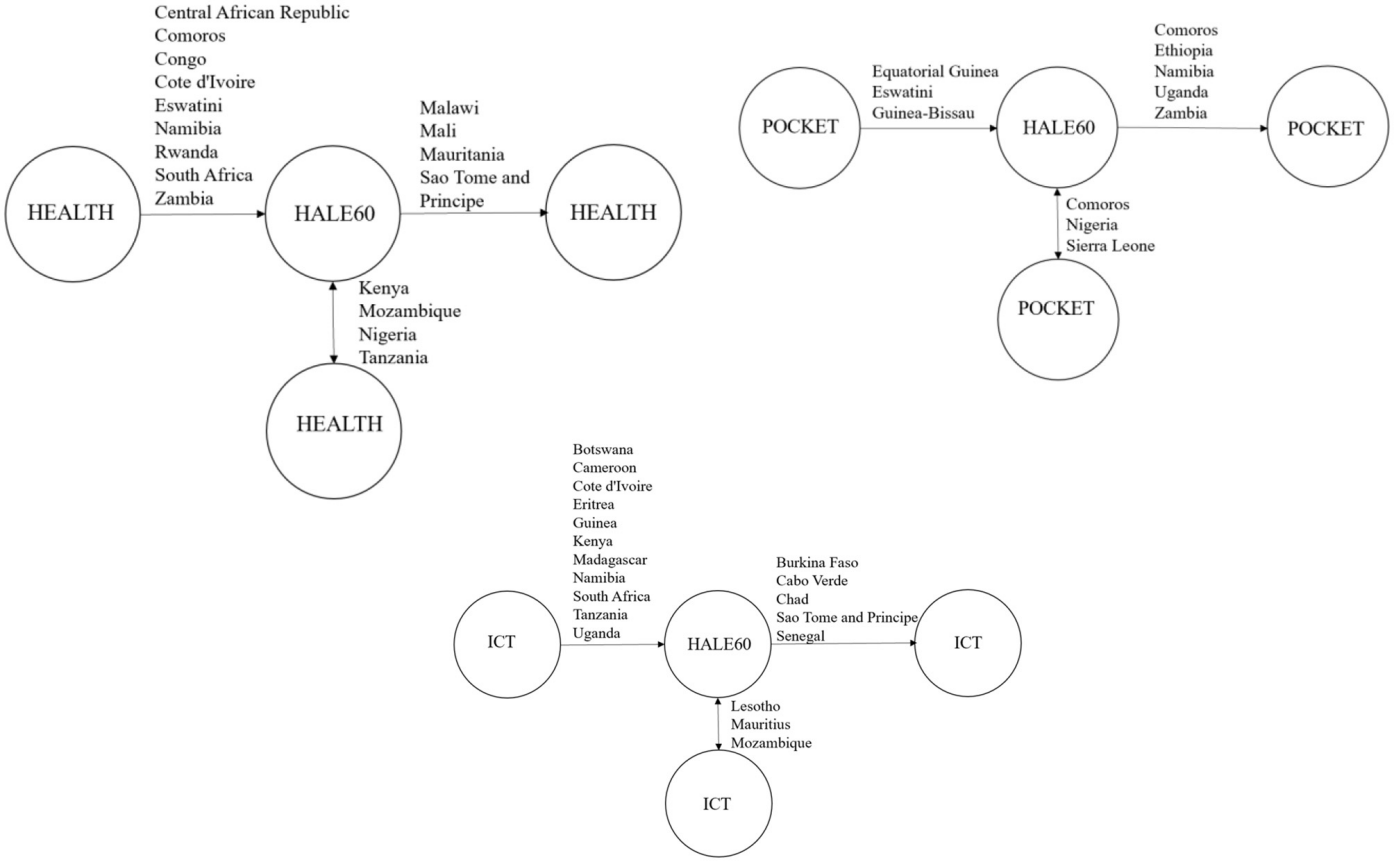
The results of the causality test among HEALTH, POCKET, ICT, and HALE60. → indicates one-way causality from … to …. while ↔ indicates a two-way causality between two variables.

Similarly, the null hypothesis is rejected for nine of 44 countries for causality from out-of-pocket expenditures to HALE60, but 7/44 stay significant after implementation of the FDR procedure. On the other hand, the null hypothesis is rejected for 12 of 44 countries for causality from HALE60 to out-of-pocket expenditures, but 10/44 stay significant after implementation of the FDR procedure. The final results are reported in Table 8 and Figure 2. In this regard, there exists a unidirectional causality from out-of-pocket expenditures to HALE60 in Equatorial Guinea, Eswatini, and Guinea-Bissau; a unidirectional causality from HALE60 to out-of-pocket expenditures in Comoros, Ethiopia, Namibia, Uganda, and Zambia; and bidirectional causality between the two variables in Botswana, Kenya, Mozambique, Nigeria, and Sierra Leone.

Finally, the null hypothesis is rejected for 17 of 44 countries for causality from ICT to HALE60, but 15/44 stay significant after implementation of the FDR procedure. On the other hand, the null hypothesis is rejected for 10 of 44 countries for causality from HALE60 to ICT, but 9/44 stay significant after the implementation of the FDR procedure. The final results are reported in Table 7. In this context, there exists a unidirectional causality from ICT to HALE60 in Botswana, Cameroon, Cote d'Ivoire, Eritrea, Guinea, Kenya, Madagascar, Namibia, South Africa, Tanzania, and Uganda and a unidirectional causality from HALE60 to ICT in Burkina Faso, Cabo Verde, Chad, Sao Tome and Principe, and Senegal and a bidirectional causality between two variables in Gambia, Lesotho, Mauritius, and Mozambique.

**Figure 3:**
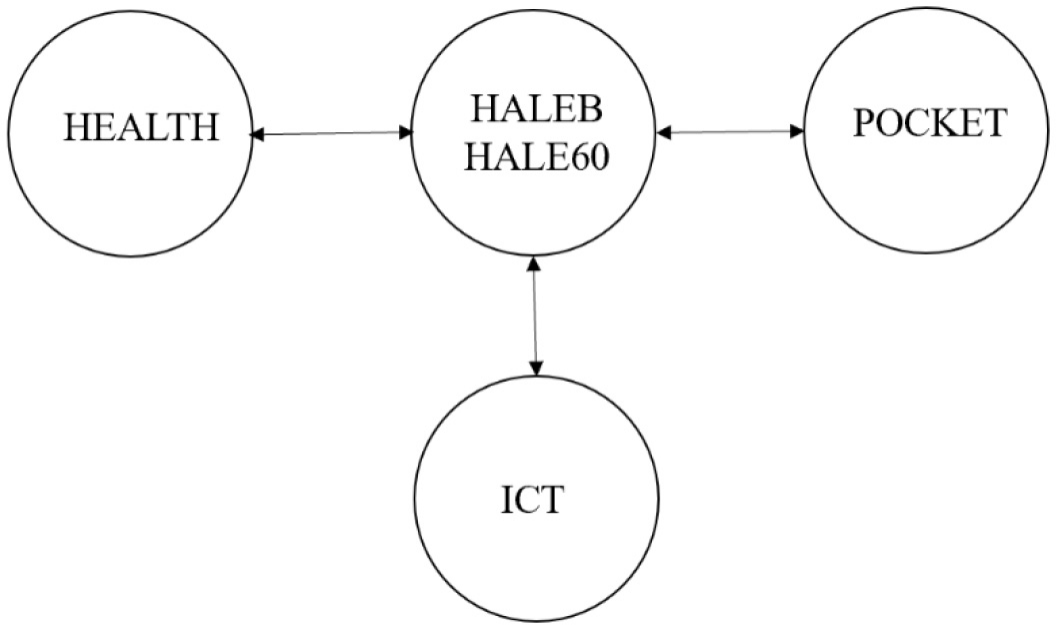
The results of panel-level causality analyses.

The causal interplay among HEALTH, POCKET, ICT, and HALEB/HALE60 is also analyzed by means of the JKS causality test for robustness, and their results are introduced in Table 9. Figure 3 also supports the results of the E-K causality test.

**Table 9:** Results of the JKS causality test.

**Hypothesis**	**HPJ statistic**
HEALTH ⇏ HALEB	12.3229^***^
HALEB ⇏ HEALTH	10.621^***^
HEALTH ⇏ HALE60	14.0677^***^
HALE60 ⇏ HEALTH	27.0918^***^
POCKET ⇏ HALEB	18.0331^***^
HALEB ⇏ POCKET	14.5649^***^
POCKET ⇏ HALE60	31.8391^***^
HALE60 ⇏ POCKET	138,243^***^
ICT ⇏ HALEB	7.0740^**^
HALEB ⇏ ICT	40.7345^***^
ICT ⇏ HALE60	7.5502^**^
HALE60 ⇏ ICT	17.2616^***^

Source: Authors own.

^***^ and ^**^ significant at 1 and 5%, respectively.

The impact of health expenditures, out-of-pocket expenditures, and ICT development on HALEB and HALE60 is also examined by means of the Driscoll–Kraay estimator, a more robust estimator for addressing the presence of autocorrelation and heteroscedasticity. Furthermore, dummy variables are used for the Ebola and COVID-19 pandemics. The estimated coefficients are presented in Table 10. The results indicate that health expenditures, out-of-pocket expenditures, and ICT development have a positive effect on both HALEB and HALE60, but the positive influence of health expenditures and out-of-pocket expenditures is relatively greater than that of ICT development. The adjusted R-square value of 0.742 indicates that the model consisting of health expenditures, out-of-pocket expenditures, and ICT development has a significant impact on HALEB and HALE60. Furthermore, the same model is estimated by the Beck–Katz estimator for the robustness of the results, and the results support the findings by the Driscoll–Kraay estimator. On the other hand, Ebola outbreaks have a negative effect on both HALEB, HALE60, while COVID-19 pandemic does not have a significant influence on HALEB and HALE60.

**Table 10:** Regression estimations.

**Independent variables**	**HALEB**		**HALE60**	
	**Driscoll–Kraay estimator**	**Beck–Katz estimator**	**Driscoll–Kraay estimator**	**Beck–Katz estimator**
	**Coefficients**	**Coefficients**	**Coefficients**	**Coefficients**
HEALTHEX	0.077^**^	0.069^**^	0.082^**^	0.071^**^
POCKET	0.065^***^	0.057^***^	0.077^**^	0.060^**^
ICT	0.028^**^	0.022^**^	0.020^**^	0.031^**^
EBOLA	−0.032^**^	−0.028^**^	−0.033^**^	−0.042^**^
COVID	0.027	0.019	0.021	0.015
C	0.964^**^	0.673^**^	1.097	1.546
Wald statistics	38.651	45.809	58.713	62.184
R-square	0.742	0.694	0.731	0.743
Adjusted R-square	0.739	0.690	0.728	0.740

^***^ and ^**^ indicates statistical significance at 1 and 5%, respectively.

## 5 Discussion

Health expenditures and out-of-pocket expenditures are among the major economic factors affecting longevity and HALE (90, 91). The increases in health expenditures are expected to positively impact HALE and lead to quantitative and qualitative improvements in health services (5, 48, 92, 93). In this regard, health expenditures and out-of-pocket expenditures can positively impact health outcomes through the progress in medical care (94). Furthermore, advances in medical technologies and wide use of these technologies can cause decreases in infant mortality rates and increases in healthy life expectancy (95, 96). Our regression results also confirm the positive effect of both health expenditures and out-of-pocket expenditures on HALEB and HALE60 suggested by these theoretical views, and they are consistent with the outcomes of Robine and Ritchie (5), Mathers et al. (45), Corris et al. (47), and Cao et al. (48).

On the other hand, the panel-level results of two causality tests point out a bidirectional causality among health expenditures, out-of-pocket expenditures, ICT, and HALEB/HALE60. These outcomes are also compatible with the theoretical views and the results of Salomon et al. (46), Corris et al. (47), Cao et al. (48), Ahmed and Almoree (51), Morina et al. (93), Novignon et al. (97), and Aytemiz et al. (98). In this context, a unidirectional causality from health expenditures to HALEB in Eritrea, Lesotho, Niger, Nigeria, Tanzania, and Zambia indicates that health expenditures rather than out-of-pocket health expenditures have significant influence on HALEB and health expenditures are largely covered by public sector. The empirical findings of Aanegola et al. (99) and Homaie Rad et al. (100) support our outcomes. Furthermore, a unidirectional causality from HALEB to health expenditures is identified in countries such as Angola, Ghana, Guinea, Malawi, Mali, and Mauritania, which are richer in natural resources, with relatively higher HALEB (6). Therefore, the increases in HALEB also raise the demand for health services in these countries. A bidirectional causality between health expenditures and HALEB is identified in Mozambique and South Africa, and this can be attributed to the remarkably higher proportion of public health expenditures than out-of-pocket expenditures in these countries (101). Finally, the insignificant effect of health expenditures on HALEB in some SSA countries can be a result of the unequal distribution and low effectiveness of health expenditures to a great extent (102).

Furthermore, a unidirectional causality from health expenditures to HALE60 is determined in Central African Republic, Comoros, Congo, Cote d'Ivoire, Eswatini, Namibia, Rwanda, South Africa, and Zambia, which is compatible with the results of Aytemiz et al. (98), Wallace (101), and Prohaska et al. (103) while a unidirectional causality from HALE60 to health expenditures is identified in Malawi, Mali, Mauritania, and Sao Tome and Principe, compatible with the results of Goldman et al. (104). The significant effect of HALE60 on health expenditures can stem from the increasing cost of longevity in the relevant countries and the importance of public health expenditures. Finally, a bidirectional causality between health expenditures and HALE60 is identified in Kenya, Mozambique, Nigeria, and Tanzania. HALE60 only increases as public health spending increases in these countries, and as HALE 60 increases, the proportion of public health spending increases (82). This situation can be explained by the insufficient number of private healthcare institutions in these countries, where out-of-pocket healthcare spending can be made.

A unidirectional causality from out-of-pocket expenditures to HALEB is identified in Botswana, Burkina Faso, Equatorial Guinea, Guinea, Malawi, Niger, and Sao Tome and Principe. This outcome shows that out-of-pocket expenditures rather than health expenditures have a significant effect on HALEB and can probably result from the inadequacy of public health services, high cost of health services, and relatively higher effectiveness of out-of-pocket expenditures in improving HALE. The results of Raeesi et al. (105) also support our results. However, a unidirectional causality from HALEB to out-of-pocket expenditures in Ethiopia, Ghana, Lesotho, Namibia, Rwanda, Senegal, Uganda, and Zambia indicates the inadequacy of health expenditures, the high cost of health services, and the necessity of out-of-pocket expenditures for health services (102), and the empirical results of Akazili et al. (106) support these outcomes. Finally, bidirectional causality between out-of-pocket expenditures and HALEB is identified in Comoros, Nigeria, and Sierra Leone.

A unidirectional causality from out-of-pocket expenditures to HALE60 is identified in Equatorial Guinea, Eswatini, and Guinea-Bissau. This result indicates that health care costs during extended old age cannot be adequately met by public health systems and that, in turn, out-of-pocket expenditures are utilized to finance the health care expenses (107, 108). On the other hand, a unidirectional causality from HALE60 to out-of-pocket expenditures, as observed in Comoros, Ethiopia, Namibia, Uganda, and Zambia, is in parallel with the theoretical assessments of Ortiz-Ospina and Roser (109) and the empirical results of Zhang et al. (108). Finally, a bidirectional causality between out-of-pocket expenditures and HALE60 is identified in Botswana, Kenya, Mozambique, Nigeria, and Sierra Leone. This bidirectional causality can be attributed to the fact that public health services do not meet expectations and that better quality health services can be obtained through out-of-pocket expenses.

The effects of ICT use on health outcomes can be varied. A number of studies discussing the benefits of ICT use on public health have concluded that ICT access and use can improve public health (107, 110). ICT use can increase health communication, provide evidence-based information flow (111), and enable extended years of life to be spent productively without disability and by actively participating in social life (51). On the other side, the negative effects of ICT on health outcomes can be associated with physiological problems such as headaches and neck pain (112), addiction, and psychological problems (76). However, regression analysis also verifies the positive effect of ICT development on HALEB and HALE60 in parallel with these theoretical considerations. Similarly, Wu and Raghupadhi (58), Raghupathi and Raghupathi (59), Alzaid et al. (60), Mimbi and Bankole (61), Majeed and Khan (62), Byaro et al. (64), Bétila (65), Megbowon and David (66), and Kouton et al. (56) unveiled a positive impact of diverse ICT indicators on LE.

In this regard, a unidirectional causality from ICT to HALEB is identified in Angola, Botswana, Central African Republic, Dem. Rep. Congo, Eritrea, Eswatini, Gambia, Lesotho, Madagascar, Mauritius, Mozambique, and Zambia. This result shows that ICT is effective on HALEB and is in line with the outcomes of Omri et al. (17), Wu and Raghupadhi (58), Alzaid et al. (60), Majeed and Khan (62), Afroz et al. (63), and Byaro et al. (64). On the other hand, a unidirectional causality from HALEB to ICT in Burkina Faso, Cabo Verde, Comoros, Congo, Equatorial Guinea, Guinea, Kenya, Sao Tome and Principe, Senegal shows that HALEB has significant impact on ICT in the relevant countries. These findings are consistent with the empirical results of Bayar et al. (68). Furthermore, a bidirectional causality between ICT and HALEB is specified in Namibia, Nigeria, and Sierra Leone. Namibia and Nigeria are among the largest economies in the SSA region, and ICT usage is relatively higher in these countries, along with Sierra Leone, than that of the other SSA countries (113). Therefore, we can conclude that ICT usage in health-seeking behavior is significant in many SSA countries.

Furthermore, a unidirectional causality from ICT to HALE60 is unveiled in Botswana, Cameroon, Cote d'Ivoire, Eritrea, Guinea, Kenya, Madagascar, Namibia, South Africa, Tanzania, and Uganda, and these outcome results can probably be attributed to easier access to health services and health-related information (114). On the other hand, a unidirectional causality from HALE60 to ICT in Burkina Faso, Cabo Verde, Chad, Sao Tome and Principe, and Senegal indicates that fact that the use of technology for accessing health services has become important among the multidimensional factors affecting the increase in healthy life expectancy (115). Finally, a bidirectional causality between ICT and HALE60 is identified in Gambia, Lesotho, Mauritius, and Mozambique.

## 6 Conclusion, limitations, and policy recommendations

A long and healthy life expectancy free from disability is a globally targeted health indicator. African countries have continued to be among the most disadvantaged countries in terms of life expectancy, although significant progress has been made in HALE worldwide. Therefore, this study investigates the nexus among health expenditure, out-of-pocket expenditure, ICT index, and HALE indicators in the SSA countries using a causality test that accounts for CD and heterogeneity over the 2000–2021 period.

This study has the following limitations:

The data period covers the 2000–2021 term because indicators of health expenditures and health life expectancy are available between 2000 and 2021.
The main limitations of the WHO HALE indicators are a lack of reliable data on mortality and morbidity, especially from low-income countries and a lack of comparability of self-reported data from health interviews and the measurement of health-state preferences for such self-reporting (116).
The study comprises 44 SSA economies and does not include Somalia, South Sudan, Sudan, and Zimbabwe due to the non-existence of data.
The main focus of this study is to analyze the nexus among indicators of health expenditures, ICT, and indicators of HALE. Therefore, the other variables affecting the HALE indicators, such as HIV/AIDS, education, income inequality, and environmental factors, structural economic, social, and health shocks, except Ebola and COVID-19, and measurement errors related to the variables, are disregarded.

The results of the causality test at the panel level uncover a two-way causality between indicators of health expenditures, ICT and HALE indicators, but the outcomes of the causality analysis at the country level differ among SSA countries. Even in countries with similar levels of economic development, different results can be seen. This difference may have many social, cultural, and political reasons. As stated in the section 5, public health expenditures may be insufficient in some countries, while in others, private sector healthcare services may surpass public healthcare services. Moreover, factors such as health-seeking culture, political stability in countries, and natural disasters, among others, can lead to differences at the country level. However, the results of the regression analysis indicate that indicators of health expenditures and ICT development positively impact HALE indicators. Furthermore, Ebola outbreaks negatively impact HALE indicators, while COVID-19 does not have a significant influence on HALE indicators. In conclusion, our outcomes highlight that both health expenditures and ICT are significant instruments to make progress in both HALEB and HALE60 in line with the associated literature.

Based on our outcomes and the relevant literature, the following policies will be useful to make achievement in a long and healthy life expectancy in the SSA countries:

Health and ICT infrastructures have not sufficiently improved in most of the SSA countries although some SSA countries are rich in terms of natural resources. Therefore, health and ICT investments should be prioritized given the significance of human capital and ICT for economic development.
On the other hand, the dominance of out-of-pocket expenditures on health in some SSA countries are mainly stemmed from significant income inequality. While those with sufficient income levels can make out-of-pocket expenditures, those without income cannot access health services that will affect their health outcomes. In this situation, public health investments and public health services become crucial. Therefore, governmental policies, through taxation and transfer payment, should be designed to decrease income inequality and equal access to healthcare services.
Finally, the share of ICT usage in the healthcare sector should be encouraged through legal and financial incentives, and training programs should be arranged to increase the digital health literacy.

Future studies can analyze the impact of income inequality and human capital on the nexus among health expenditures, ICT, and HALE indicators.

## Data Availability

The original contributions presented in the study are included in the article/supplementary material, further inquiries can be directed to the corresponding author.
